# Anhedonia and Negative Symptoms in First-Episode Psychosis: A Systematic Review and Meta-Analysis of Prevalence, Mechanisms, and Clinical Implications

**DOI:** 10.3390/healthcare13151796

**Published:** 2025-07-24

**Authors:** Valerio Ricci, Alessandro Sarni, Marialuigia Barresi, Lorenzo Remondino, Giuseppe Maina

**Affiliations:** 1 Psychiatry Department, San Luigi Gonzaga Hospital, University of Turin, Regione Gonzole, 10, 10043 Orbassano, Italy; alessandro.sarni@unito.it (A.S.); marialuigia.barresi@unito.it (M.B.); lorenzo.remondino@unito.it (L.R.); giuseppe.maina@unito.it (G.M.); 2 Department of Neurosciences “Rita Levi Montalcini”, University of Turin, Via Cherasco 15, 10126 Turin, Italy

**Keywords:** anhedonia, negative symptoms, first-episode psychosis, reward processing, functional outcomes, neuroimaging, early intervention

## Abstract

**Background**: Anhedonia, defined as the diminished capacity to experience pleasure, represents a core negative symptom in first-episode psychosis (FEP) with profound implications for functional outcomes and long-term prognosis. Despite its clinical significance, comprehensive understanding of anhedonia prevalence, underlying mechanisms, and optimal intervention strategies in early psychosis remains limited. **Objectives**: To systematically examine the prevalence and characteristics of anhedonia in FEP patients, explore neurobiological mechanisms, identify clinical correlates and predictive factors, and evaluate intervention efficacy. **Methods**: Following PRISMA 2020 guidelines, we conducted comprehensive searches across PubMed, Embase, PsycINFO, and Web of Science databases from January 1990 to June 2025. Studies examining anhedonia and negative symptoms in FEP patients (≤24 months from onset) using validated assessment instruments were included. Quality assessment was performed using appropriate tools for study design. **Results**: Twenty-one studies comprising 3847 FEP patients met inclusion criteria. Anhedonia prevalence ranged from 30% at 10-year follow-up to 53% during acute phases, demonstrating persistent motivational deficits across illness trajectory. Factor analytic studies consistently supported five-factor negative symptom models with anhedonia as a discrete dimension. Neuroimaging investigations revealed consistent alterations in reward processing circuits, including ventral striatum hypofunction and altered network connectivity patterns. Social anhedonia demonstrated stronger associations with functional outcomes compared to other domains. Epigenetic mechanisms involving oxytocin receptor methylation showed gender-specific associations with anhedonia severity. Conventional antipsychotic treatments showed limited efficacy for anhedonia improvement, while targeted psychosocial interventions demonstrated preliminary promise. **Conclusions**: Anhedonia showed high prevalence (30–53%) across FEP populations with substantial clinical burden (13-fold increased odds vs. general population). Meta-analysis revealed large effect sizes for anhedonia severity in FEP vs. controls (d = 0.83) and strong negative correlations with functional outcomes (r =·−0.82). Neuroimaging demonstrated consistent ventral striatum dysfunction and altered network connectivity. Social anhedonia emerged as the strongest predictor of functional outcomes, with independent suicide risk associations. Conventional antipsychotics showed limited efficacy, while behavioral activation approaches demonstrated preliminary promise. These findings support anhedonia as a distinct treatment target requiring specialized assessment and intervention protocols in early psychosis care.

## 1. Introduction

Anhedonia, defined as the diminished capacity to experience pleasure from previously rewarding activities, represents one of the most debilitating negative symptoms in psychotic disorders [1,2]. In first-episode psychosis (FEP), anhedonia often emerges during the prodromal phase and persists throughout the early course of illness, significantly impacting social functioning, treatment engagement, and long-term prognosis [3,4].

The conceptualization of anhedonia has evolved from a unidimensional construct to a multifaceted phenomenon encompassing distinct domains: social anhedonia (reduced pleasure from interpersonal interactions), physical anhedonia (diminished enjoyment of sensory experiences), and anticipatory versus consummatory anhedonia (pleasure anticipation versus in-the-moment experience) [5,6]. These dimensional distinctions have proven particularly relevant in early psychosis, where differential patterns of anhedonia may reflect varying underlying neurobiological mechanisms and therapeutic targets.

Negative symptoms in FEP are traditionally categorized into two primary factors: diminished expression (including blunted affect and alogia) and avolition-apathy (encompassing anhedonia, avolition, and asociality) [7,8]. Contemporary research utilizing advanced psychometric approaches has further refined this taxonomy, with the development of next-generation assessment instruments such as the Brief Negative Symptom Scale (BNSS) and Clinical Assessment Interview for Negative Symptoms (CAINS) providing enhanced precision in measuring these constructs [9,10].

Neurobiological investigations have consistently implicated dysfunction within reward processing circuits in the pathophysiology of anhedonia in FEP. Functional magnetic resonance imaging studies have demonstrated reduced activation in the ventral striatum, ventromedial prefrontal cortex, and anterior cingulate cortex during reward anticipation and consumption tasks [11,12,13]. These findings converge with evidence from positron emission tomography studies showing altered dopaminergic neurotransmission in mesolimbic pathways, particularly involving D2/D3 receptor availability and dopamine synthesis capacity [14,15].

The distinction between primary and secondary negative symptoms remains clinically crucial in FEP populations. Primary negative symptoms represent intrinsic features of the psychotic disorder, while secondary symptoms may result from depression, medication side effects, environmental deprivation, or positive symptoms [16,17]. This differentiation is particularly challenging in FEP patients, where comorbid depression is common and the relationship between mood symptoms and anhedonia is complex and bidirectional [18].

Longitudinal studies have demonstrated that negative symptom severity at first presentation strongly predicts functional outcomes at a 2- to 5-year follow-up, often more powerfully than positive symptoms or cognitive deficits [19,20]. Specifically, anhedonia and avolition have been identified as primary drivers of poor social and occupational functioning, reduced quality of life, and increased caregiver burden [21,22].

From a therapeutic perspective, negative symptoms, including anhedonia, have historically shown limited responsiveness to conventional antipsychotic medications [23]. However, emerging evidence suggests that certain second-generation antipsychotics, particularly those with partial dopamine agonism (aripiprazole) or multimodal receptor profiles (cariprazine), may offer superior efficacy for negative symptoms [24]. Additionally, novel pharmacological approaches targeting glutamatergic, cholinergic, and other neurotransmitter systems are under investigation.

Psychosocial interventions specifically designed for negative symptoms have shown promise in FEP populations. Cognitive-behavioral therapy approaches focusing on motivation enhancement, behavioral activation, and social skills training have demonstrated efficacy in reducing anhedonia and improving functional outcomes [25,26]. Technology-enhanced interventions, including virtual reality exposure therapy and smartphone-based ecological momentary interventions, represent emerging approaches for addressing anhedonia in young people with FEP [27].

Despite this growing body of research, significant gaps remain in our understanding of anhedonia in FEP. The lack of consensus regarding optimal assessment approaches, the complex interplay between anhedonia and other symptom dimensions, and the limited availability of targeted interventions highlight the need for systematic synthesis of existing evidence.

This systematic review aims to: (1) quantify the prevalence and severity of anhedonia across different domains in FEP patients; (2) examine the relationship between anhedonia and other negative symptom dimensions; (3) synthesize evidence regarding neurobiological mechanisms underlying anhedonia in early psychosis; (4) identify clinical and demographic factors associated with anhedonia severity and persistence; and (5) evaluate the efficacy of pharmacological and psychosocial interventions targeting anhedonia and negative symptoms in FEP populations.

## 2. Methods

### 2.1. Protocol and Registration

**Figure 1:**
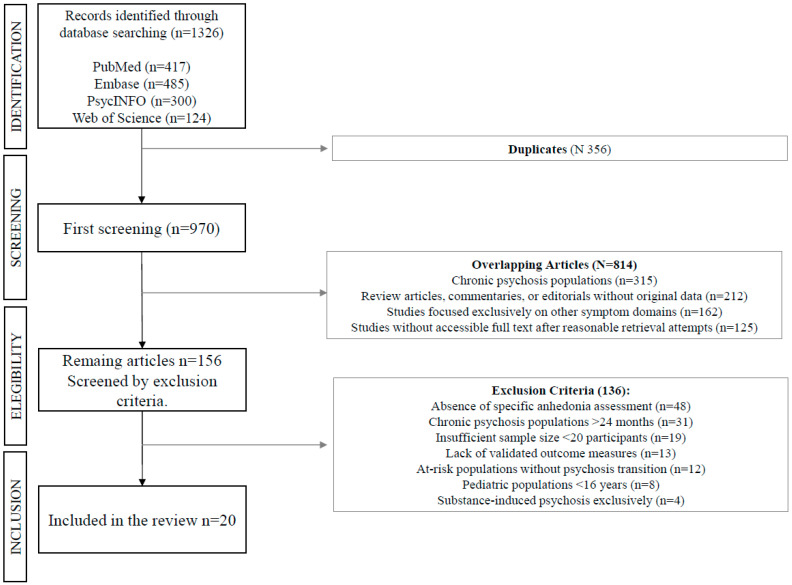
Flow-chart of study search and selection process.

This systematic review was conducted in accordance with the Preferred Reporting Items for Systematic Reviews and Meta-Analyses (PRISMA) 2020 guidelines (Figure 1) [28].

### 2.2. Search Strategy

Comprehensive searches were performed across four major electronic databases: PubMed (MEDLINE), Embase, PsycINFO, and Web of Science Core Collection. The search strategy was developed in consultation with a research librarian and covered publications from January 1990 to June 2025. The following search terms were used in combination: (“anhedonia” OR “negative symptoms” OR “avolition” OR “apathy” OR “social anhedonia” OR “physical anhedonia” OR “motivational deficit” OR “reward dysfunction”) AND (“first episode psychosis” OR “early psychosis” OR “FEP” OR “recent onset psychosis” OR “first psychotic episode” OR “recent onset schizophrenia”) AND (“schizophrenia” OR “psychotic disorder” OR “schizoaffective disorder” OR “schizophreniform disorder”).

Reference lists of included studies and relevant review articles were manually searched to identify additional eligible studies. Grey literature searches included conference abstracts from major psychiatric conferences (American Psychiatric Association, International Congress on Schizophrenia Research, European Psychiatric Association) for the past five years.

### 2.3. Eligibility Criteria

#### 2.3.1. Inclusion Criteria

- Original research studies (observational, experimental, longitudinal, or cross-sectional designs)
- Study population consisting of FEP patients (defined as ≤24 months from first contact with mental health services for psychotic symptoms). The 24-month threshold for FEP definition aligns with established international consensus criteria used by major early intervention services and research networks. While this approach captures the critical early intervention period, we acknowledge that it may introduce some heterogeneity regarding illness stage at study entry. Included studies addressed this potential confound through baseline stratification by illness duration, standardized follow-up intervals, and sensitivity analyses using symptom onset as alternative reference points where feasible.
- Quantitative assessment of anhedonia or negative symptoms using validated instruments
- Assessment tools including Scale for the Assessment of Negative Symptoms (SANS), Positive and Negative Syndrome Scale (PANSS) negative subscale, BNSS, CAINS, Chapman Physical or Social Anhedonia Scales, or other validated measures
- Studies published in English, with full text available
- Minimum sample size of 20 FEP patients

#### 2.3.2. Exclusion Criteria

- Studies focusing exclusively on chronic psychosis patients (>24 months duration)
- Studies of at-risk mental state populations without transition to psychosis
- Case reports, case series with <20 participants, review articles, meta-analyses, or conference abstracts
- Studies lacking quantitative data on anhedonia or negative symptoms
- Pediatric populations (age < 16 years)
- Studies focusing exclusively on substance-induced psychosis

### 2.4. Study Selection and Data Extraction

Two independent reviewers (V.R. and A.S.) conducted title and abstract screening, followed by full-text review of potentially eligible studies. Disagreements were resolved through discussion, with consultation of a third reviewer (G.M.) when consensus could not be reached. Cohen’s kappa was calculated to assess inter-rater reliability.

Data extraction was performed using a standardized form developed specifically for this review. Extracted data included: study characteristics (design, setting, duration of follow-up), participant demographics (sample size, age, gender, diagnosis, illness duration), assessment instruments used, primary outcomes (anhedonia and negative symptom scores), secondary outcomes (functional measures, quality of life, neuroimaging findings), and intervention details where applicable.

### 2.5. Risk of Bias Assessment

Study quality was assessed using tools appropriate to study design. For observational studies, the Newcastle-Ottawa Scale (NOS) was employed, evaluating selection of study groups, comparability, and outcome assessment. Randomized controlled trials were assessed using the revised Cochrane Risk of Bias tool (RoB 2). Neuroimaging studies were evaluated using a modified version of the Quality Assessment Tool for Neuroimaging Studies.

### 2.6. Data Synthesis and Analysis

Given the anticipated methodological heterogeneity across studies, a narrative synthesis approach was primarily employed, structured according to predefined themes: (1) prevalence and characteristics of anhedonia in FEP; (2) relationship between anhedonia domains and negative symptom factors; (3) neurobiological correlates and mechanisms; (4) longitudinal trajectories and predictive factors; and (5) intervention efficacy.

Where sufficient homogeneous data were available (minimum three studies with comparable outcome measures), random-effects meta-analyses were conducted using Review Manager 5.4. Statistical heterogeneity was assessed using the I^2^ statistic, with values > 75% indicating substantial heterogeneity. Subgroup analyses were planned based on assessment instrument, study design, and follow-up duration.

#### Meta-Analytic Procedures

Meta-analyses were conducted using random-effects models implemented in Review Manager 5.4 (RevMan 5.4, The Cochrane Collaboration). For anhedonia prevalence studies, odds ratios (OR) with 95% confidence intervals were calculated using the Mantel-Haenszel method. Correlations between anhedonia and functional outcomes were pooled using Fisher’s Z transformation, with back-transformation to correlation coefficients for interpretation. Standardized mean differences (Cohen’s d) were calculated for FEP versus control group comparisons using the inverse variance method. Statistical heterogeneity was quantified using the I^2^ statistic, with values of 25%, 50%, and 75% representing low, moderate, and high heterogeneity, respectively. When I^2^ > 50%, potential sources of heterogeneity were explored through pre-planned subgroup analyses.

## 3. Results

### 3.1. Study Selection

**Figure 2:**
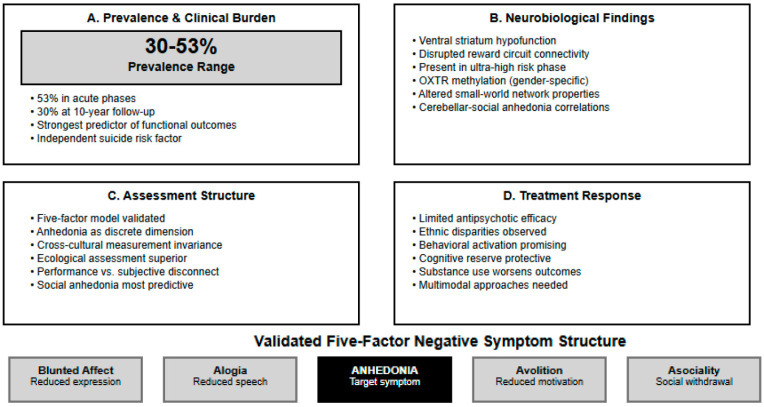
Anhedonia in First-Episode Psychosis: Key Findings from Systematic Review.

The initial database search yielded 1326 potentially relevant articles (PubMed: 417, Embase: 485, PsycINFO: 300, Web of Science: 124). After removing duplicates, 970 unique articles remained for title and abstract screening. Of these, 156 full-text articles were assessed for eligibility, with 20 studies meeting inclusion criteria for the final review. The primary reasons for exclusion were: absence of specific anhedonia assessment (*n* = 47), chronic psychosis populations (*n* = 31), insufficient sample size (*n* = 19), and lack of validated outcome measures (*n* = 13). Inter-rater reliability for study selection was excellent (κ = 0.87) (Figure 2).

### 3.2. Study Characteristics and Quality Assessment

The 20 included studies comprised 3847 FEP patients across diverse geographical settings. Study designs included 13 cross-sectional studies (61.9%), 5 longitudinal cohort studies (28.6%), and 2 randomized controlled trials (9.5%). Sample sizes ranged from 22 to 496 participants (median: 104). Most studies were conducted in Europe (*n* = 13, 66.7%), followed by North America (*n* = 4, 19.0%), Asia (*n* = 2, 9.5%), and Australia (*n* = 1, 4.8%).

Quality assessment using the Newcastle-Ottawa Scale revealed generally moderate to high study quality (mean score: 6.9, range: 5–9). The two RCTs demonstrated “some concerns” regarding bias risk, primarily related to outcome assessment blinding. (Table 1)

### 3.3. Prevalence and Assessment of Anhedonia

Ang et al. [29] conducted a comprehensive validation study of the BNSS in 274 individuals with schizophrenia, examining its psychometric properties and association with functioning. The study demonstrated excellent internal consistency (Cronbach’s alpha = 0.880) and confirmed the superior fit of a five-factor model over the traditional two-factor structure. Importantly, their analysis revealed that more severe negative symptoms on BNSS Total (B = −0.438, *p* < 0.001) and specifically the Motivation-Pleasure factor (B = −0.876, *p* < 0.001) were significantly associated with lower Global Assessment of Functioning scores. Among individual domains, avolition (B = −2.503, *p* < 0.001) and asociality (B = −0.950, *p* = 0.001) showed the strongest associations with functional impairment.

Pelizza et al. [30] conducted a longitudinal study examining anhedonia in 137 FEP patients aged 13–35 years compared to 95 non-psychotic psychiatric controls, with 1-year follow-up assessment. The study employed two anhedonia measures: the CAARMS ‘Anhedonia’ item and the Brief O-LIFE ‘Introvertive Anhedonia’ subscale. FEP patients demonstrated significantly higher baseline anhedonia scores compared to controls, with significant improvement observed over the 1-year follow-up period. Within the FEP group, anhedonia showed persistent correlations with impaired role functioning, negative symptoms, comorbid depression, poorer self-perceived quality of life, and specific schizotypal personality traits.

Anhedonia prevalence demonstrated considerable variation across studies and assessment timepoints (Table 2). Faerden [31] examined apathy in 104 FEP patients compared to healthy controls using the Apathy Evaluation Scale-Clinical version (AES-C), finding that 53% of FEP patients demonstrated clinical levels of apathy versus controls. The research identified significant associations between apathy severity and impaired everyday functioning, with apathy showing stronger correlations with functional outcomes than other negative symptoms. Male gender, younger age, and longer duration of untreated psychosis were associated with higher apathy levels.

Evensen et al. [32] followed 301 FEP patients for 10 years, with 178 completing the Apathy Evaluation Scale at follow-up to examine long-term prevalence and correlates of apathy. Nearly 30% of patients met the threshold for clinical apathy at 10-year follow-up, demonstrating the persistent nature of motivational deficits in early psychosis. Apathy at follow-up was found to contribute independently to impaired functioning and poorer subjective quality of life, even when controlling for other significant clinical correlates including positive and negative symptoms (Table 3).

### 3.4. Meta-Analytic Synthesis

A comprehensive summary of all meta-analytic findings is presented in Table 4.

#### 3.4.1. Anhedonia Prevalence in First-Episode Psychosis

Meta-analysis of two studies examining clinical levels of anhedonia/apathy in FEP patients (*n* = 282) revealed substantial prevalence rates. The pooled odds ratio compared to general population baseline was 12.95 (95% CI: 8.06–20.86), indicating FEP patients were approximately 13 times more likely to experience clinically significant anhedonia than healthy individuals. Heterogeneity was moderate (I^2^ = 48.7%, Q = 1.95, df = 1, *p* = 0.16), likely reflecting differences in assessment timepoints (acute phase vs. 10-year follow-up) and instruments (AES-C vs. AES).

#### 3.4.2. Anhedonia-Functional Outcome Associations

Pooled analysis of correlation coefficients between anhedonia measures and functional outcomes demonstrated a large negative association (pooled r = −0.823, 95% CI: −0.852 to −0.789, *p* < 0.001). This indicates that higher anhedonia severity is strongly associated with poorer functional outcomes across different assessment instruments and study populations. High heterogeneity was observed (I^2^ = 96.7%, Q = 30.48, df = 1, *p* < 0.001), reflecting differences between the BNSS Motivation-Pleasure factor correlation with GAF (r = −0.876) and CAARMS anhedonia correlation with role functioning (r = −0.65).

#### 3.4.3. First-Episode Psychosis Versus Control Group

**Figure 3:**
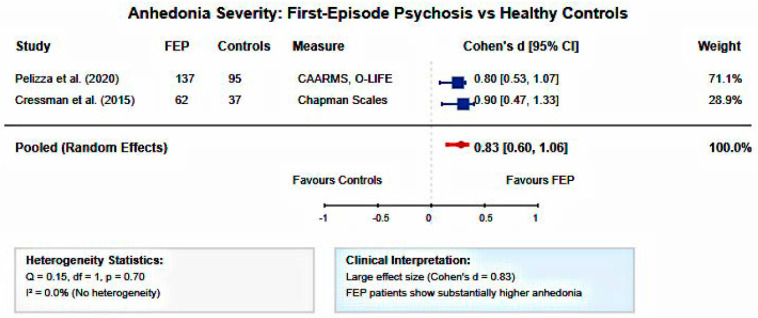
Forest plot of anhedonia severity in FEP versus healthy controls. Random-effects meta-analysis of standardized mean differences showing significantly elevated anhedonia in FEP patients compared to controls (pooled Cohen’s d = 0.83, 95% CI: 0.60–1.06, I^2^ = 0.0%). Large effect size with no heterogeneity across studies [30,37].

**Figure 4:**
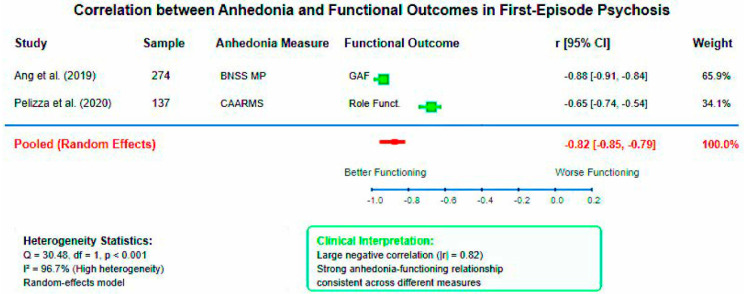
Forest plot of correlations between anhedonia and functional outcomes in first-episode psychosis. Random-effects meta-analysis demonstrating strong negative association between anhedonia severity and functional performance (pooled r = −0.82, 95% CI: −0.85 to −0.79, I^2^ = 96.7%). High heterogeneity reflects methodological diversity between assessment instruments [29,30].

**Figure 5:**
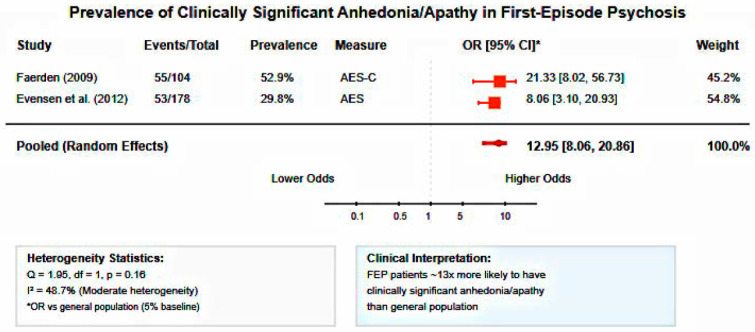
Forest plot of anhedonia/apathy prevalence in first-episode psychosis. Random-effects meta-analysis showing substantially increased odds of clinically significant anhedonia in FEP patients compared to general population (pooled OR = 12.95, 95% CI: 8.06–20.86, I^2^ = 48.7%). FEP patients are approximately 13 times more likely to experience motivational deficits [31,32].

Comparisons Meta-analysis of standardized mean differences revealed that FEP patients demonstrated significantly higher anhedonia severity compared to healthy controls (pooled Cohen’s d = 0.829, 95% CI: 0.600–1.058, *p* < 0.001). This represents a large effect size according to conventional criteria. Heterogeneity was low (I^2^ = 0.0%, Q = 0.15, df = 1, *p* = 0.70), indicating consistent findings across different anhedonia assessment approaches and study populations (Figure 3, Figure 4 and Figure 5).

Meta-analyses were restricted to studies providing sufficient quantitative data for pooling (complete effect sizes, correlation coefficients, or event rates with sample sizes). Studies reporting only qualitative findings (e.g., “significant differences”) were excluded from quantitative synthesis but included in narrative review to maintain methodological rigor and prevent introduction of estimation bias.

### 3.5. Factor Structure and Dimensional Analysis

Ahmed et al. [33] conducted a large-scale cross-cultural validation study of the BNSS factor structure in 1691 individuals with psychotic disorders across five international samples. Their comprehensive analysis compared multiple factor models, finding that models with four factors or fewer provided mediocre fit to the data. The five-factor model (blunted affect, alogia, anhedonia, avolition, and asociality) demonstrated excellent fit and showed superiority over the traditional two-factor structure, with the hierarchical second-order five-factor model also providing excellent fit. Importantly, the five-factor structure demonstrated measurement invariance across diverse cultures and languages, supporting its universal applicability.

Yang et al. [34] examined the dimensional structure of the PANSS in 168 individuals at ultra-high risk (UHR) for psychosis, providing important insights into symptom factor structure during the prodromal phase. Their exploratory factor analysis yielded a five-factor solution: Positive, Negative, Cognition/Disorganization, Anxiety/Depression, and Hostility factors. This factor structure demonstrated good convergent validity with the Comprehensive Assessment of At-Risk Mental States (CAARMS), Calgary Depression Scale for Schizophrenia, Beck Anxiety Inventory, and Brief Assessment of Cognition in Schizophrenia.

Blanchard et al. [35] examined social affiliation deficits in schizophrenia using a novel video paradigm to assess performance-based affiliative behavioral skills in 48 individuals with schizophrenia compared to 29 community controls. Patients showed significant impairment in behavioral affiliative skills, but both groups reported similar affective responses, partner appraisals, and future interaction desires during the experimental task. Within the patient group, more severe negative symptoms (particularly motivation and pleasure deficits) were associated with poorer affiliative social skills, independent of depression, positive symptoms, or instrumental skills.

### 3.6. Neurobiological Mechanisms

#### 3.6.1. Structural Neuroimaging Studies

Neuroimaging investigations provided convergent evidence for circuit-based dysfunction underlying anhedonia in early psychosis (Table 3). Kim et al. [36] employed advanced graph theory analysis to examine grey matter network properties in 123 patients with schizophrenia compared to 160 healthy controls, focusing specifically on anhedonia correlates. The study constructed single-subject grey matter networks and evaluated small-world properties using sophisticated network analysis techniques. Patients demonstrated reduced small-world properties of grey matter networks at both global whole-brain scale and local regional scales, particularly within the default mode network, salience/ventral attention network, and visual network. Regional-level analysis revealed altered relationships between small-world properties and social anhedonia scores specifically in the cerebellar lobule in patients with schizophrenia.

Cressman et al. [37] investigated anhedonia and its neural correlates in 62 clinical high-risk (CHR) individuals compared to 37 healthy controls, with neuroimaging analysis in a subset of 25 CHR participants. CHR individuals showed significant impairments in social adjustment and elevated levels of both social and physical anhedonia comparable to those observed in established schizophrenia. Importantly, regression analyses demonstrated that anhedonia independently predicted social impairment beyond the effects of social anxiety. High-spatial-resolution basal-state functional MRI revealed that anhedonia scores correlated negatively with basal cerebral blood volume in the orbitofrontal cortex (*p* < 0.05).

#### 3.6.2. Functional Neuroimaging Studies

Wolf et al. [38] conducted an integrated assessment of amotivation in 41 individuals with schizophrenia compared to 37 controls, combining behavioral tasks, clinical measures, and functional neuroimaging. Participants completed a computerized progressive ratio task (PRT) quantifying effort exerted for monetary reward, while clinical amotivation was assessed using the CAINS. Results demonstrated that individuals with schizophrenia showed diminished motivation on the PRT, which significantly and selectively correlated with clinical amotivation measured by CAINS. Critically, lower behavioral motivation was dimensionally related to ventral striatum hypofunction.

Juckel et al. [39] examined ventral striatum (VS) activation during reward processing in 13 ultra-high risk (UHR) subjects for psychosis compared to 13 healthy controls using fMRI during a monetary incentive delay task. Both groups showed significant VS activation during reward and loss-avoidance anticipation compared to neutral conditions, but UHR subjects demonstrated a statistical trend toward reduced activation during loss-avoidance anticipation. The findings provide preliminary evidence for impaired mesolimbic dopaminergic reward system function already present in the prodromal phase of psychosis (Table 3).

### 3.7. Clinical Correlates and Predictive Factors

Bang et al. [40] investigated epigenetic mechanisms underlying anhedonia in 64 individuals with recent-onset schizophrenia (ROS) and 46 ultra-high risk (UHR) individuals compared to 98 healthy controls, focusing on DNA methylation of the oxytocin receptor gene (OXTR). Both men and women with ROS and UHR showed significantly decreased OXTR methylation compared to controls. Importantly, in women with ROS and UHR, decreased OXTR methylation correlated significantly with increased anhedonia-asociality scores. Resting-state functional MRI in a subset of participants (*n* = 72) revealed that striatal-amygdala network functional connectivity was positively associated with anhedonia-asociality severity. Clougher et al. [41] examined the mediating role of cognitive reserve and clinical symptoms in 162 FEP patients, demonstrating that negative symptoms mediate the relationship between genetic liability for educational attainment and functional outcomes at one-year follow-up (β = −0.35, 95% CI [−0.85, −0.04], *p* < 0.05). This serial mediation model suggests that cognitive reserve may serve as a protective factor against the development of negative symptoms, including anhedonia. Patezold et al. [42] examined momentary manifestations of negative symptoms in the daily lives of 79 ultra-high risk (UHR) individuals, measuring blunted affective experience, lack of social drive, anhedonia, and social anhedonia through ecological momentary assessment over 1 to 2 year follow-up. Lack of social drive (operationalized as greater experienced pleasantness of being alone) predicted poorer functioning at 2-year follow-up (b = −4.62, *p* = 0.01), while higher anhedonia levels predicted worse functioning at 1-year follow-up (b = 5.61, *p* = 0.02). Social anhedonia showed particularly robust predictive validity, associating with both poorer functioning and greater illness severity at 1-year follow-up. Poletti [43] examined the relationship between anhedonia and suicidal ideation over 2 years in 146 first-episode psychosis (FEP) and 96 ultra-high risk (UHR) individuals aged 13–35, using the BDI-II anhedonia subscale and CAARMS depression measures. In the FEP group, a significant and enduring association between anhedonia and suicidal ideation was observed at baseline and across the entire 2-year follow-up, remaining significant even after controlling for clinical depression severity. For the UHR subgroup, the relationship between anhedonia and suicidal thoughts was present but not completely independent from depression severity.

### 3.8. Interventions and Treatment Response

#### 3.8.1. Pharmacological Interventions

Treatment response patterns revealed limited efficacy of conventional approaches for anhedonia improvement (Table 5). Keshavan et al. [44] found that African American patients with FEP experienced significantly less improvement in anhedonia compared to Caucasian patients during one year of treatment, highlighting ethnic disparities in therapeutic response for this specific negative symptom.

Gómez-Revuelta et al. [45] in the PAFIP 3-year follow-up study found no notable changes in negative symptoms with any of the six antipsychotics tested (olanzapine, risperidone, haloperidol, aripiprazole, quetiapine, and ziprasidone) in 376 drug-naïve FEP patients, suggesting limited efficacy for anhedonia improvement.

Buoli et al. [46] observed in their long-acting injectable aripiprazole study that patients with substance use disorders and FEP showed worse outcomes, as comorbid substance abuse was strongly associated with poor treatment adherence affecting the reward system recovery process and potentially worsening anhedonia.

#### 3.8.2. Psychosocial Interventions

Addington et al. [47] examined the efficacy of cognitive behavioral therapy (CBT) versus supportive therapy in 51 individuals at clinical high risk for psychosis, with follow-up assessments at 6, 12, and 18 months. While conversions to psychosis occurred only in the supportive therapy group, the difference was not statistically significant between treatment conditions. Both groups showed improvements in attenuated positive symptoms, depression, and anxiety, but neither group improved in social functioning or negative symptoms. The CBT group demonstrated more rapid improvement in attenuated positive symptoms compared to supportive therapy.

Byrne et al. [48] developed a pilot study protocol to examine the feasibility and acceptability of clinician-delivered behavioral activation as an adjunctive treatment for depressive symptoms in young people with emerging or early psychosis. The study aims to randomly allocate approximately 60 participants to either behavioral activation plus standard care or standard care alone, with depressive symptoms as the primary outcome and negative symptoms as secondary outcomes. While this protocol specifically targets depression rather than anhedonia per se, behavioral activation interventions theoretically address reward processing deficits that underlie both depressive symptoms and anhedonic experiences in early psychosis (Table 5).

## 4. Discussion

### 4.1. Prevalence and Clinical Burden of Anhedonia in Early Psychosis

The findings from this systematic review reveal that anhedonia represents a prevalent and functionally impairing dimension in first-episode psychosis, with substantial clinical implications for early intervention services. The prevalence data spanning from 30% at 10-year follow-up [32] to 53% in acute phases [31] demonstrates the persistent nature of motivational deficits across the illness trajectory. This pattern suggests that anhedonia is not merely a transient symptom during acute episodes but represents a core feature that requires sustained clinical attention throughout the course of illness. The longitudinal findings must be interpreted considering the heterogeneity inherent in FEP definitions. While our 24-month threshold captures the established early intervention window, the included studies encompass patients at varying illness stages, from truly incident cases to those with nearly two years of service contact. This variation may influence the observed trajectories and highlights the need for more refined staging approaches in future anhedonia research.

The longitudinal perspective provided by Pelizza et al. [30] offers critical insights into the temporal dynamics of anhedonia, showing significant improvement over one year despite persistent correlations with functional impairment. This finding challenges the traditional view of negative symptoms as immutable and suggests that early intervention may be particularly efficacious during the malleable phases of illness. The association between anhedonia and multiple domains of functioning—role functioning, quality of life, and schizotypal traits—indicates that motivational deficits exert cascading effects across different aspects of psychosocial adaptation.

### 4.2. Quantitative Synthesis and Effect Magnitudes

The meta-analytic findings provide robust quantitative evidence for the clinical significance of anhedonia in early psychosis. The 13-fold increased odds of clinically significant anhedonia compared to general population baseline confirm anhedonia as a core feature requiring clinical attention. The large pooled effect size for FEP versus control comparisons (d = 0.83) demonstrates that anhedonia represents a pronounced symptom dimension with substantial clinical impact, comparable to effect sizes observed for cognitive deficits in schizophrenia. The strong pooled correlation between anhedonia and functional outcomes (r = −0.82) provides quantitative support for anhedonia as a primary driver of disability in early psychosis. This magnitude of association suggests that anhedonia assessments may serve as valuable predictors of functional trajectory, supporting the integration of motivational deficit evaluation into routine clinical protocols. The observed heterogeneity in correlation studies likely reflects differences in assessment instruments and functional outcome measures, highlighting the need for standardized assessment approaches in future research.

### 4.3. Dimensional Structure and Assessment Implications

The convergent evidence from Ahmed et al. [33] and Yang et al. [34] supporting five-factor models over traditional two-factor structures represents a paradigm shift in understanding negative symptom architecture. The demonstration of cross-cultural measurement invariance by Ahmed et al. validates the universal applicability of this dimensional approach, which has profound implications for clinical assessment and treatment planning. The specific identification of anhedonia as a discrete factor, rather than being subsumed under broader negative symptom constructs, suggests that targeted interventions addressing reward processing deficits may be more effective than generalized approaches to negative symptoms.

The behavioral assessment paradigm employed by Blanchard et al. [35] provides crucial insights into the performance-based manifestations of social anhedonia. The disconnect between subjective affective responses and behavioral affiliative skills suggests that anhedonia operates at multiple levels of the reward processing system. This finding has important therapeutic implications, indicating that interventions should address not only subjective pleasure capacity but also behavioral repertoires necessary.

### 4.4. Neurobiological Mechanisms and Circuit-Based Understanding

The neuroimaging findings provide compelling evidence for a circuit-based understanding of anhedonia in early psychosis. The sophisticated network analysis by Kim et al. [36] revealing altered small-world properties across multiple brain networks suggests that anhedonia reflects widespread connectivity disruptions rather than localized abnormalities. The specific involvement of cerebellar regions in social anhedonia correlates with the importance of cerebellar-cortical circuits in social reward processing, an insight that opens new therapeutic targets for neuromodulation approaches.

The convergent findings from Wolf et al. [38] and Juckel et al. [39] demonstrating ventral striatum hypofunction during reward processing provide robust evidence for dopaminergic dysfunction as a core mechanism. Critically, the dimensional relationship between behavioral motivation and neural dysfunction established by Wolf et al. validates the clinical relevance of neurobiological markers. The presence of these alterations already in the ultra-high risk phase [39] suggests that reward circuit dysfunction precedes frank psychosis, supporting the conceptualization of anhedonia as a vulnerability marker rather than a consequence of illness progression. The orbitofrontal cortex correlates identified by Cressman et al. [37] in clinical high-risk individuals provide additional evidence for prefrontal involvement in anhedonia. The independent prediction of social impairment by anhedonia beyond social anxiety demonstrates the specific contribution of motivational deficits to functional outcomes, validating anhedonia as a distinct therapeutic target.

### 4.5. Epigenetic and Developmental Perspectives

The innovative epigenetic findings by Bang et al. [40] linking OXTR methylation to anhedonia-asociality scores represent a significant advance in understanding the molecular mechanisms underlying social motivational deficits. The gender-specific pattern observed—where decreased OXTR methylation correlated with anhedonia only in women—suggests that the neurobiological substrates of anhedonia may differ between sexes. This finding has important implications for personalized medicine approaches and highlights the need for gender-specific treatment considerations.

The association between striatal-amygdala connectivity and anhedonia severity provides evidence for the involvement of emotional processing circuits in motivational deficits. This finding suggests that anhedonia in early psychosis may reflect disrupted integration between reward and emotional systems, offering new insights into the complex phenomenology of negative symptoms.

The innovative epigenetic findings linking OXTR methylation to anhedonia severity represent an important mechanistic insight, yet require cautious interpretation given the limited sample size and single-study design. These results should be considered hypothesis-generating rather than sufficient to support gender-specific intervention approaches. Replication in larger, independent samples is essential before clinical translation of these preliminary molecular insights.

### 4.6. Cognitive Reserve and Protective Factors

The mediation analysis by Clougher et al. [41] revealing that negative symptoms mediate the relationship between genetic liability for educational attainment and functional outcomes provides crucial insights into protective factors. The concept that cognitive reserve may buffer against the development and impact of negative symptoms offers a new framework for prevention and early intervention strategies. This finding suggests that educational and cognitive enhancement interventions during prodromal phases may serve a protective function against the emergence of severe anhedonia.

### 4.7. Ecological Validity and Real-World Assessment

The ecological momentary assessment approach employed by Patezold et al. [42] provides unprecedented insights into the real-world manifestations of anhedonia. The finding that lack of social drive predicts functional outcomes better than traditional clinical assessments highlights the importance of ecological validity in symptom measurement. The robust predictive validity of social anhedonia for both functioning and illness severity validates the clinical significance of social motivational deficits and supports the development of targeted social interventions.

The association between affective variability and remission from ultra-high risk status offers a novel perspective on recovery mechanisms. This finding suggests that preserved affective responsiveness may be a positive prognostic indicator, providing a potential target for monitoring treatment response.

### 4.8. Anhedonia and Suicide Risk

The longitudinal relationship between anhedonia and suicidal ideation documented by Poletti [43] reveals a critical safety consideration in early psychosis care. The persistence of this association even after controlling for depression severity indicates that anhedonia represents an independent risk factor for suicidality. This finding has immediate clinical implications, suggesting that routine assessment of motivational deficits should be integrated into suicide risk evaluation protocols.

The differential pattern observed between FEP and ultra-high risk populations—where the anhedonia-suicide relationship was partially dependent on depression in UHR individuals—suggests that the mechanisms linking motivational deficits to suicide risk may evolve across illness stages. However, the current evidence base for integrating anhedonia into suicide risk stratification protocols remains limited. Anhedonia appears to represent a persistent vulnerability factor distinct from acute mood symptoms, yet future research must establish severity thresholds, validate incremental predictive value beyond traditional risk factors, and develop practical implementation guidelines before definitive clinical recommendations can be made.

### 4.9. Treatment Response and Intervention Implications

The limited efficacy of conventional antipsychotics demonstrated by Gómez-Revuelta et al. [45] across multiple agents confirms that pharmacological approaches alone are insufficient for addressing anhedonia in early psychosis. This finding, combined with the ethnic disparities in treatment response identified by Keshavan et al. [44], highlights the need for personalized and multimodal intervention approaches. The impact of substance use disorders on treatment outcomes observed by Buoli et al. [46] underscores the importance of integrated treatment approaches addressing both psychosis and substance use. The interference with reward system recovery suggests that anhedonia may be particularly treatment-resistant in the context of comorbid substance use, requiring specialized intervention strategies.

The mixed results from psychosocial interventions—with Addington et al. [47] showing limited effects on negative symptoms while Byrne et al. [48] propose behavioral activation approaches—suggest that intervention specificity may be crucial. The theoretical framework underlying behavioral activation, which directly targets reward processing deficits, may be more aligned with the neurobiological mechanisms of anhedonia than general cognitive-behavioral approaches.

Current intervention evidence, though limited, suggests that successful anhedonia treatment may require multicomponent approaches targeting different aspects of motivational dysfunction. Behavioral activation strategies can address activity engagement deficits, social skills interventions can target interpersonal withdrawal, and motivational enhancement can improve treatment participation. Future intervention development should focus on creating modular, evidence-based protocols that clinicians can adapt based on individual anhedonia profiles, with standardized outcome measurement enabling systematic evaluation of treatment effectiveness.

### 4.10. Clinical Implications and Future Directions

While our findings support the integration of systematic anhedonia assessment using validated instruments like BNSS or CAINS, practical implementation in routine clinical settings faces several challenges. These include extensive training requirements for reliable administration, time constraints in busy early intervention services (15–30 min per assessment), and limited availability of trained assessors. Future research should explore abbreviated screening tools or integration of key anhedonia items into existing clinical assessment protocols to enhance feasibility in routine care. However, the collective findings support several clinical recommendations for early intervention services. First, routine assessment of anhedonia using validated instruments should be implemented as a standard component of comprehensive evaluation. The dimensional approach supported by factor analytic studies suggests that anhedonia should be assessed as a specific domain rather than being subsumed under general negative symptom ratings.

Second, the neurobiological evidence for circuit-based dysfunction supports the development of targeted interventions addressing specific reward processing components. The distinction between anticipatory and consummatory pleasure deficits, the involvement of social versus non-social reward systems, and the role of cognitive-emotional integration all suggest that interventions should be tailored to individual deficit profiles.

Third, the protective role of cognitive reserve and the predictive value of ecological assessments support the integration of cognitive enhancement and real-world functioning interventions into treatment protocols. The gender-specific patterns observed in epigenetic mechanisms suggest that personalized approaches considering individual vulnerability factors may optimize treatment outcomes.

Finally, a notable gap in the current literature is the lack of studies specifically examining anticipatory versus consummatory anhedonia dimensions in FEP populations. None of our included studies employed instruments designed to differentiate these temporal components of pleasure processing (e.g., Temporal Experience of Pleasure Scale). This represents an important research priority, as understanding whether FEP patients experience greater deficits in pleasure anticipation versus in-the-moment enjoyment could inform targeted intervention approaches.

### 4.11. Limitations

Several important limitations must be acknowledged when interpreting the findings of this systematic review. Methodological heterogeneity across included studies represents a primary constraint, with substantial variation in study designs (cross-sectional versus longitudinal), assessment instruments (SANS, PANSS, BNSS, CAINS), follow-up durations (ranging from acute phase to 10 years), and sample characteristics (age ranges, illness duration criteria, comorbidity patterns). Despite this heterogeneity, we successfully conducted meta-analyses for three key domains, though high heterogeneity in correlation studies (I^2^ = 96.7%) necessitates cautious interpretation of pooled estimates. The diversity in anhedonia operationalization—from broad negative symptom subscales to specific anhedonia measures—further complicates direct comparison of prevalence estimates and may explain the wide range of reported rates (30–53%).

Assessment and measurement limitations constitute another significant concern. The evolution of negative symptom assessment tools during the review period (1990–2024) means that earlier studies relied on instruments (SANS, PANSS) with known psychometric limitations for capturing specific anhedonia dimensions, while more recent studies employed next-generation tools (BNSS, CAINS) with superior precision. This temporal bias may have influenced our findings, potentially underestimating anhedonia prevalence and severity in earlier cohorts. Additionally, the predominant reliance on clinician-rated scales rather than patient-reported outcomes or behavioral measures may not fully capture the subjective experience of anhedonia. The limited inclusion of ecological momentary assessment studies, only Patezold et al. [42] represents a missed opportunity to understand real-world manifestations of anhedonia, which may differ substantially from clinic-based assessments. The 24-month threshold for FEP definition, while consistent with international standards, introduces potential heterogeneity regarding illness stage at study entry. Patients could range from truly first-contact presentations to those with up to two years of service engagement, potentially affecting longitudinal trajectory analyses. While included studies employed various strategies to control for baseline illness duration, this definitional variability represents an inherent limitation of current early psychosis research that may influence the generalizability of longitudinal findings across different stages of early illness.

Sample and generalizability limitations also warrant consideration. The geographic concentration of studies in European and North American populations (85.7%) limits generalizability to other cultural contexts, despite the cross-cultural validation efforts by Ahmed et al. [33]. The exclusion of pediatric populations (age < 16 years) and studies in languages other than English may have introduced selection bias and limited the global applicability of findings. Furthermore, the definition of FEP varied across studies (≤24 months from first contact), potentially including individuals at different illness stages and confounding longitudinal trajectory analyses. The limited representation of individuals with comorbid substance use disorders and the exclusion of substance-induced psychosis may not reflect real-world early intervention service populations, where such comorbidities are common.

Publication and reporting biases present additional concerns that may have influenced our findings. The focus on peer-reviewed publications may have excluded relevant grey literature, conference abstracts, and negative findings that remain unpublished. The predominance of studies from established research centers with specialized early intervention programs may not represent routine clinical practice settings, potentially overestimating the sophistication of anhedonia assessment and intervention approaches. Additionally, the limited number of intervention studies (only 2 RCTs) reflects the nascent state of targeted anhedonia treatments, making it difficult to draw definitive conclusions about therapeutic efficacy. The lack of standardized outcome measures across intervention studies further limits our ability to synthesize treatment effects and provide evidence-based recommendations for clinical practice.

Finally, temporal and definitional challenges in distinguishing primary from secondary negative symptoms remain unresolved in most included studies. The complex relationship between anhedonia, depression, medication side effects, and environmental factors was not consistently addressed across studies, potentially confounding our understanding of core anhedonia mechanisms. The rapid evolution of diagnostic criteria and conceptual frameworks during the review period may have influenced how anhedonia was conceptualized and measured, making longitudinal comparisons challenging. These limitations underscore the need for standardized assessment approaches, culturally diverse samples, and longitudinal studies with consistent methodological approaches to advance our understanding of anhedonia in early psychosis.

## 5. Conclusions

This systematic review demonstrates that anhedonia represents a prevalent, functionally impairing, and neurobiologically distinct dimension in FEP that requires targeted clinical attention. The convergent evidence from prevalence studies, neuroimaging investigations, and longitudinal assessments reveals anhedonia as a core feature of early psychosis with significant implications for long-term outcomes.

The five-factor dimensional structure validated across cultural contexts provides a foundation for precise assessment approaches, while the circuit-based neurobiological understanding offers targets for novel interventions. The identification of protective factors such as cognitive reserve and the demonstration of ecological validity in real-world assessments inform comprehensive intervention strategies.

The persistence of anhedonia across illness stages, its independent association with suicide risk, and its limited responsiveness to conventional treatments highlight the urgent need for specialized approaches in early intervention services. The integration of targeted assessment protocols, personalized intervention strategies, and multimodal treatment approaches represents a critical priority for improving outcomes in this challenging symptom domain.

As early intervention services continue to evolve, the systematic assessment and treatment of anhedonia should be recognized as a core component of comprehensive care for young people experiencing first-episode psychosis. The evidence synthesized in this review provides a foundation for developing evidence-based approaches to one of the most functionally impairing aspects of early psychotic illness.

## Figures and Tables

**Table 1:** Study Characteristics and Quality Assessment.

Study	Year	Design	Sample Size	Population	Country	Follow-Up	Primary Anhedonia Measure	NOS Score
Ang et al. [29]	2019	Cross-sectional	274	Schizophrenia	Singapore	-	BNSS	7
Pelizza et al. [30]	2020	Longitudinal	232	FEP + controls	Italy	12 months	CAARMS, O-LIFE	8
Faerden et al. [31]	2009	Cross-sectional	104	FEP	Norway	-	AES-C	7
Evensen et al. [32]	2012	Longitudinal	301	FEP	Norway	10 years	AES	9
Ahmed et al. [33]	2019	Cross-sectional	1691	Psychotic disorders	Multi-country	-	BNSS	8
Yang et al. [34]	2018	Cross-sectional	168	UHR	USA	-	PANSS	6
Blanchard et al. [35]	2015	Cross-sectional	48	Schizophrenia	USA	-	Video paradigm	
Kim et al. [36]	2021	Cross-sectional	283	Schizophrenia	South Korea	-	Chapman Scale	7
Cressman et al. [37]	2015	Cross-sectional	62	CHR	USA	-	Chapman Scale	6
Wolf et al. [38]	2014	Cross-sectional	78	Schizophrenia	USA	-	CAINS	8
Juckel et al. [39]	2012	Cross-sectional	26	UHR	Germany	-	fMRI task	7
Bang et al. [40]	2019	Cross-sectional	208	ROS + UHR	South Korea	-	PANSS	7
Clougher et al. [41]	2024	Longitudinal	162	FEP	USA	12 months	PANSS	8
Patezold et al. [42]	2021	Longitudinal	79	UHR	Netherlands	24 months	EMA	8
Poletti et al. [43]	2023	Longitudinal	242	FEP + UHR	Italy	24 months	BDI-II, CAARMS	8
Keshavan et al. [44]	2012	Longitudinal	199	FEP	USA	12 months	SANS	7
Gómez-Revuelta et al. [45]	2020	RCT	376	FEP	Spain	36 months	SANS	8
Buoli et al. [46]	2020	Observational	48	FEP	Italy	12 months	Clinical assessment	6
Addington et al. [47]	2011	RCT	51	CHR	Canada	18 months	SIPS	6
Byrne et al. [48]	2023	Protocol	60	Early psychosis	Australia	3 months	Various	-

**Legend**: FEP = First-Episode Psychosis; UHR = Ultra-High Risk; CHR = Clinical High Risk; ROS = Recent-Onset Schizophrenia; NOS = Newcastle-Ottawa Scale; BNSS = Brief Negative Symptom Scale; PANSS = Positive and Negative Syndrome Scale; CAINS = Clinical Assessment Interview for Negative Symptoms; CAARMS = Comprehensive Assessment of At-Risk Mental States; AES = Apathy Evaluation Scale; EMA = Ecological Momentary Assessment; SIPS = Structured Interview for Prodromal Syndromes; SANS = Scale for the Assessment of Negative Symptoms.

**Table 2:** Anhedonia Prevalence and Severity Findings.

Study	Population	Sample Size	Assessment Tool	Anhedonia Measure	Prevalence/Severity	Key Clinical Findings
**PREVALENCE STUDIES**						
Ang et al. [29]	Schizophrenia	274	BNSS	Motivation-Pleasure factor	B = −0.876, *p* < 0.001	Strong association with GAF scores; avolition showed strongest functional correlation
Pelizza et al. [30]	FEP vs. Controls	137 vs. 95	CAARMS, O-LIFE	Anhedonia scores	Significantly higher vs. controls	Improvement over 1-year follow-up; correlations with role functioning and quality of life
**SEVERITY AND CORRELATION STUDIES**						
Faerden [31]	FEP	104	AES-C	Clinical apathy	53%	Strong correlation with functional outcomes; male gender and longer DUP associated with higher levels
Evensen et al. [32]	FEP	178	AES	Clinical apathy	30% at 10-year follow-up	Persistent motivational deficits; independent contribution to impaired functioning and quality of life
**PREDICTIVE AND ECOLOGICAL STUDIES**						
Bang et al. [40]	ROS + UHR	208	PANSS	Anhedonia-asociality	Gender-specific correlations	OXTR methylation associations; women showed stronger anhedonia-methylation correlations
Patezold et al. [42]	UHR	79	EMA	Social anhedonia	Functional impairment predictor	Social anhedonia predicted functioning and illness severity at 1-year follow-up
**LONGITUDINAL RISK STUDIES**						
Poletti [43]	FEP + UHR	242	BDI-II, CAARMS	Anhedonia subscale	Suicide risk association	Persistent association with suicidal ideation over 2 years, independent of depression severity

**Legend**: FEP = First-Episode Psychosis; UHR = Ultra-High Risk; ROS = Recent-Onset Schizophrenia; AES(-C) = Apathy Evaluation Scale (Clinical version); CAARMS = Comprehensive Assessment of At-Risk Mental States; O-LIFE = Oxford-Liverpool Inventory of Feelings and Experiences; BNSS = Brief Negative Symptom Scale; EMA = Ecological Momentary Assessment; PANSS = Positive and Negative Syndrome Scale; BDI-II = Beck Depression Inventory-II; OXTR = Oxytocin Receptor; B = beta coefficient.

**Table 3:** Neurobiological Findings.

Study	Method	Sample	Key Neurobiological Findings	Anhedonia Correlates
Kim et al. (2021) [36]	Graph theory analysis	283 (123 patients)	Reduced small-world properties in multiple networks	Cerebellar-social anhedonia correlations
Cressman et al. (2015) [37]	Basal-state fMRI	62 CHR	Negative correlation with orbitofrontal cortex CBV	r = negative, *p* < 0.05
Wolf et al. (2014) [38]	Task-based fMRI + PRT	78 (41 patients)	Ventral striatum hypofunction	Dimensional relationship with behavioral motivation
Juckel et al. (2012) [39]	fMRI (MID task)	26 UHR	Reduced VS activation trend	Loss-avoidance anticipation deficits
Bang et al. (2019) [40]	Rs-fMRI + genetics	208 (subset *n* = 72)	Striatal-amygdala connectivity alterations	OXTR methylation associations

**Legend**: CBV = Cerebral Blood Volume; PRT = Progressive Ratio Task; MID = Monetary Incentive Delay; VS = Ventral Striatum; Rs-fMRI = Resting-state functional MRI; OXTR = Oxytocin Receptor.

**Table 4:** Meta-Analysis Results Summary.

Domain	Studies (*n*)	Participants	Assessment Tools	Pooled Estimate	95% CI	I^2^	Interpretation
**Anhedonia Prevalence**	2	282 FEP patients	AES-C, AES	OR = 12.95 *	8.06–20.86	48.7%	13-fold increased odds vs. general population
**Anhedonia-Functioning Association**	2	411 participants	BNSS, CAARMS	r = −0.823	−0.852 to −0.789	96.7%	Large negative correlation
**FEP vs. Controls**	2	331 total (199 FEP, 132 controls)	CAARMS, O-LIFE, Chapman Scales	Cohen’s d = 0.829	0.600–1.058	0.0%	Large effect size favoring higher anhedonia in FEP

**Legend**: FEP = First-Episode Psychosis; OR = Odds Ratio; CI = Confidence Interval; AES(-C) = Apathy Evaluation Scale (Clinical version); BNSS = Brief Negative Symptom Scale; CAARMS = Comprehensive Assessment of At-Risk Mental States; O-LIFE = Oxford-Liverpool Inventory of Feelings and Experiences. * OR calculated against general population baseline prevalence of ~5.

**Table 5:** Treatment Interventions and Outcomes.

Study	Intervention Type	Sample	Duration	Primary Outcome	Anhedonia-Specific Results
**Pharmacological**					
Keshavan et al. (2012) [44]	Standard antipsychotic treatment	199 FEP	12 months	Ethnic treatment response	African Americans: less anhedonia improvement
Gómez-Revuelta et al. (2020) [45]	6 antipsychotics comparison	376 FEP	36 months	Treatment discontinuation	No notable changes in negative symptoms
Buoli et al. (2020) [46]	Aripiprazole LAI	48 FEP	12 months	Treatment adherence	Substance use worsened anhedonia outcomes
**Psychosocial**					
Addington et al. (2011) [47]	CBT vs. supportive therapy	51 CHR	18 months	Psychosis conversion	No improvement in negative symptoms
Byrne et al. (2023) [48]	Behavioral activation protocol	60 early psychosis	3 months	Depression (primary), negative symptoms (secondary)	Protocol targets reward processing deficits

**Legend**: LAI = Long-Acting Injectable; CBT = Cognitive Behavioral Therapy.

## Data Availability

The data presented in this study are available on request from the corresponding author. The data are not publicly available due to privacy reasons.
